# Accuracy in osteotomy drilling using a new universal and disposable drill-stop device for dental implant drills: an *in vitro* study using a bovine rib model

**DOI:** 10.4317/medoral.26495

**Published:** 2024-04-14

**Authors:** Fabio Camacho-Alonso, Mario Pérez-Sayáns, Jesús Pato-Mourelo, Juan Enrique Martínez-Martínez

**Affiliations:** 1 DDS, PhD. Department of Oral Surgery, University of Murcia, Murcia, Spain; 2 DDS, PhD. Department of Oral Medicine, Oral Surgery and Implantology, University of Santiago de Compostela, Spain MedOralRes Group, Health Research Institute of Santiago de Compostela (IDIS). Santiago de Compostela, Spain; 3 DDS, PhD. Private dental practice, Galicia, Spain; 4 DDS, PhD. Private dental practice, Murcia, Spain

## Abstract

**Background:**

This study aimed to evaluate the surgical accuracy of a new universal disposable stop system for implant drills (FCA Universal Drill Stop).

**Material and Methods:**

A total of 60 bovine ribs were included in this *in vitro* study. The ribs were randomized into three study groups (*n*=20 ribs per group). In each study group (Group1: drills without stop or control group, Group 2: prefabricated drills with stop or gold standard group, and Group 3: drills with FCA Universal Drill Stop) a total of 100 osteotomies were performed with implant drills in each group, following the drilling sequence for the placement of a dental implant of 10 mm length and 4 mm diameter. The accuracy of the depth of the osteotomies was quantified clinically (with periodontal probe) and radiologically, using ImageJ version 1.48v software.

**Results:**

The order of highest to lowest accuracy (clinical and radiological) in the depth of osteotomies was: FCA Universal Drill Stop> prefabricated drills with a stop>drills without stop, with statistically significant differences being observed between both systems with stop with respect to the control group, although not between them.

**Conclusions:**

The new universal disposable stop system for implant drills, offers similar accuracy to prefabricated drills with stop, with both systems being much more accurate than implant drills without stop. Although this experimental evaluation showed favourable results, further clinical studies are necessary.

** Key words:**Drill stop, dental implant drill, universal, disposable, osteotomies.

## Introduction

Tooth loss is one of the biggest problems affecting oral health, interfering with quality of life and causing the patient to have a deficient functional capacity, leading to nutritional, aesthetic and psychological alterations. These alterations, based on changes in facial morphology after tooth loss, are irreversible and can only be solved by making a correct dental prosthesis (1,2).

In recent decades, oral implantology has shown that oral rehabilitation of patients with single, multiple, or total dental loss is a predicTable treatment with a high success rate in the short, medium, and long term, because dental implant prostheses allow correct functionality and offer great comfort to the patient as their design does not require mucosal support or excessive extension over intraoral soft tissues. In fact, the current longer life expectancy has made the loss of one or more teeth more frequent, which has led to a significant increase in the use of dental implants; to such an extent that it is estimated that 19% of the world's population aged over 45 years have undergone some type of treatment involving dental implants (3,4).

This increased use of dental implants for the aesthetic and functional rehabilitation of tooth loss increases the risk of iatrogenic injury. In this regard, to ensure the correct performance of osteotomies in the maxilla and to carry out safe and effective implant treatments, it is essential that clinicians receive surgical training in implant dentistry, as well as adequate pre-surgical planning (5). There are numerous factors that can influence the development of an incorrect ostectomy: radiological discrepancies in the pre-surgical study, factors associated to the implantologist (anxiety, lack of practice, errors in planning), and factors associated to the patient (nervousness that can cause involuntary movements, saliva or blood that impede the visibility of the depth marks on the surgical drills, and having low bone density that facilitates over-drilling due to lack of resistance from the bone) (6).

Poor surgical training or lack of anatomical knowledge of the maxillary structures, poor implant planning or any of the above-mentioned factors associated with the implantologist or patient can lead to incorrect osteotomies with the risk of causing iatrogenic injuries to the maxilla or mandible. In the maxilla, the main anatomical structures that can be injured during dental implant surgery are the maxillary sinuses (in the posterior maxillary region) and the nostrils (in the anterior maxillary region). As for odontogenic maxillary sinusitis related to implant treatments, it occurs at a relatively low incidence rate (0.05%), however, it continues to increase every year (7), and may be due to an initial injury with the implant drills on the Schneiderian membrane or an initial or late displacement of the dental implant within the maxillary sinus due to surgical errors (8). In the anterior region of the upper maxilla, care must also be taken with the height of the maxillary bone when performing implant osteotomies, as this area is bounded by the nostrils. The cortical bone of the nostrils is harder than the maxillary bone, so there will be some resistance when drilling in this area. When the cortical bone, and therefore the nasal mucosa, which is very thin, is perforated and the dental implant is placed, the patient will feel discomfort after surgery. In addition, if the mucosa is perforated during surgery, it may interfere with airflow (9). In the mandible, a surgical error in implant osteotomies may cause temporary or irreversible dysaesthesia, hypoaesthesia or paraesthesia due to injury to the inferior dental nerve or the inferior alveolar nerve in the mandibular posterior region, the mandibular canal and mental foramen and the anterior loop of the mental nerve, the incisor nerve (10), and even injury to the terminal branches of the sublingual and submental arteries in the interforaminal region (11). Finally, an error in implant osteotomies in the mandible can lead to perforation of the cortical bone in the mandibular and sublingual fossae (12).

To avoid iatrogenic injuries and to achieve a precise drilling depth during implant osteotomies, numerous drill stop systems are available, which provide safety, efficiency and precision, better visibility and reduced stress for both clinicians and patient. Numerous dental implant brands (Nobel Biocare®, Kolten, Switzerland; Biomet 3i®, Florida, USA; Astra Tech Dental Implant System®, Dentsply, Sirona, Sweden; etc) now offer drill stops that are sized specifically for twist drills that correspond to the implant widths of their companies. Most these company-specific stop systems are reusable, but their main drawback is the difficulty of cleaning them, since they are small devices. To avoid this problem, there is a disposable stop system for drills, although it is also specific for implant drills manufactured by this company (Straumann®, Villeret, Switzerland), and even some commercial companies that manufacture their own prefabricated drills with stops (Nueva Galimplant® S.L.U., Lugo, Spain) without the need to incorporate any additional device to the drills. However, none of these systems is universal, and although some years ago a universal reusable stop device (Zosseo®, Seattle, USA) was marketed which could fit drills from multiple companies, it is not currently available (9).

To date, although the advantages of using implant drill stops for precise maxillary osteotomies are well known, only 11% of clinicians use these devices. Although the reason why their use in dental implant surgeries is so low is unknown, it could be due to economic reasons (high cost of these devices), lack of universality and difficulty in achieving adequate cleaning of these devices although they are reusable (13-15). Therefore, this study aimed to evaluate the surgical accuracy of a new disposable universal stop system for implant drills (FCA Universal Drill Stop).

## Material and Methods

- In vitro study design

The entire study was developed following the recommendations of the Checklist for Reporting In-vitro Studies (CRIS) (16). The study protocol was approved by the Biosafety Committee on Experimentation of the University of Murcia (401/2021) and was conducted between May 2021 and February 2022 in the University Dental Clinic and the Research Support Service of the University of Murcia. To calculate a representative sample size, a power of 80% was required (5% alpha level). The bovine ribs were obtained from a total of 3 cows aged 18 months and weighing 650 kg, since the use of bovine ribs as a model for *in vitro* studies has been widely described in the scientific literature, due to their similarity to human maxillary bones because of their close resemblance in the proportion of cortical bone and cancellous bone (17).

To standardise the bone samples so that they were all homogeneous, we obtained a total of 78 ribs from the 3 animals (each cow has a total of 13 pairs of ribs, *n*=26 ribs for each animal). The proximal and distal regions of each rib were removed due to their curvature, leaving us with a central portion of approximately 15 centimetres. To homogenise the samples, we used the following inclusion criteria: ≥15 mm in length, ≥6 mm in height, and presence of between 1.5 and 2 mm of cortical bone, following the bovine rib standardisation methodology proposed by Strbac *et al*., (18). Of the 78 bovine ribs, 60 were included in the study because they met the inclusion criteria.

Fresh bovine specimens were cleaned with physiological saline and residual soft tissues were removed. Subsequently, they were immersed in a solution of physiological saline and ethanol 1:1 (Sigma-Aldrich Chemistry, S.A., Madrid, Spain), following the methodology proposed by Tricio *et al*., (19). To minimise thermophysiological changes and mechanical properties of the samples, the ribs were frozen in physiological saline (Sigma-Aldrich Chemistry, S.A., Madrid, Spain) at -10° C (20). Before osteotomies were performed, the samples were thawed, kept at a temperature of ±21° C for 3 hours wrapped in gauze soaked in physiological saline (Sigma-Aldrich Chemistry, S.A., Madrid, Spain). Finally, before performing the implantological osteotomies, each specimen was placed on a polymerised silicone base (Labosil®, Protechno S.A., Girona, Spain) to facilitate drilling.

- Study groups according to type of osteotomies

Randomisation was performed using an online service www.ranomization.com. The 60 bovine ribs were randomised into three groups (*n*=20 per group). Each rib ≥15 cm in length was sectioned into 5 blocks of ≥3 cm in length and each block was placed on a silicone base as described above to facilitate the drilling sequence of implant osteotomies in each study group. All study groups were drilled to an osteotomy depth of 10 mm and following the manufacturer's instructions were drilled for a Type II bone at 800-1100 rpm with saline irrigation (to ensure the best possible milling). The drilling sequence (all milling were made by the same experienced operator) was: 2 mm diameter initial drill and 4 posterior helical drills of 2, 2.6, 3.2 and 3.6 mm diameter (Nueva Galimplant S.L.U., Lugo, Spain).

**Figure 1:**
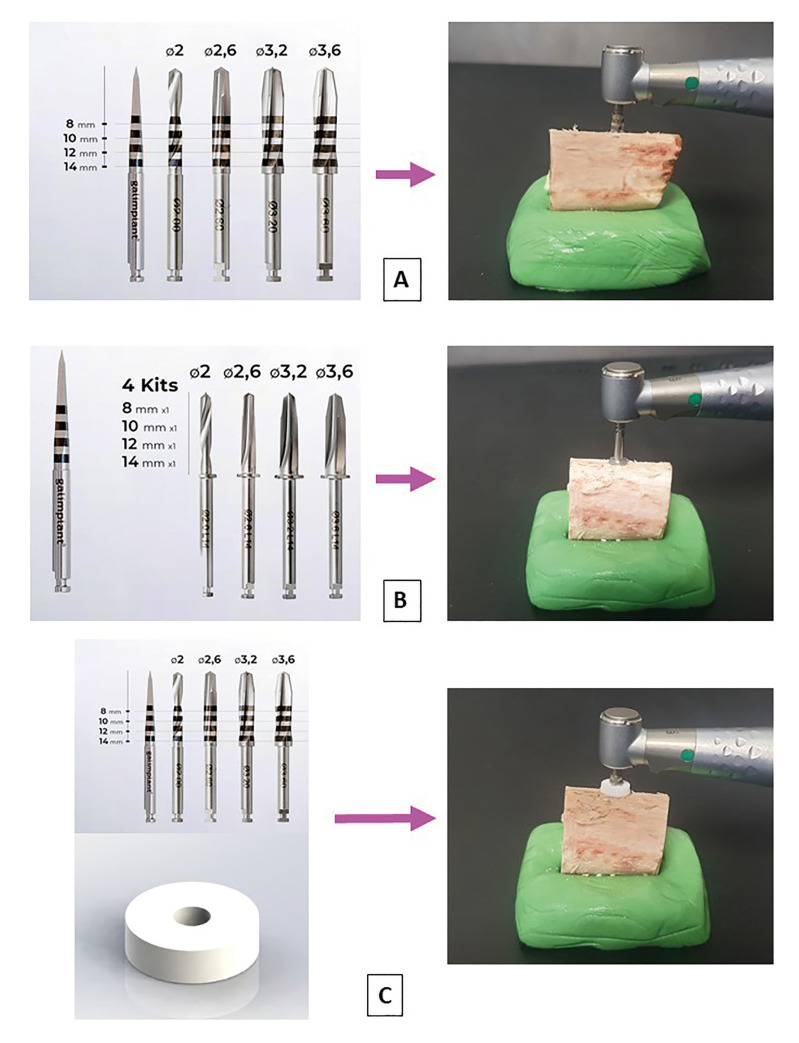
Study groups according to the type of osteotomies. The drilling sequence was: start drill of 2 mm diameter and 4 posterior helical drills of 2; 2,6; 3,2 and 3.6 mm diameter. A: Group 1 (drilling without stops). B: Group 2 (drilling with prefabricated drills with stops). C: Group 3 (drilling with the new universal and disposable device for dental implant drills FCA Universal Drill Stop).

Group 1 (drilling without stops, *n*=100), we perform the complete drilling sequence of a 10 mm long and 4 mm wide IPX Galimplant® (Nueva Galimplant S.L.U., Lugo, Spain) to place it in a juxtaosseous position (Fig. 1).

Group 2 (drilling with prefabricated drills with stops, *n*=100) (Nueva Galimplant® S.L.U., Lugo, Spain) without the need to fit any additional device to the drills (Fig. 1).

**Figure 2:**
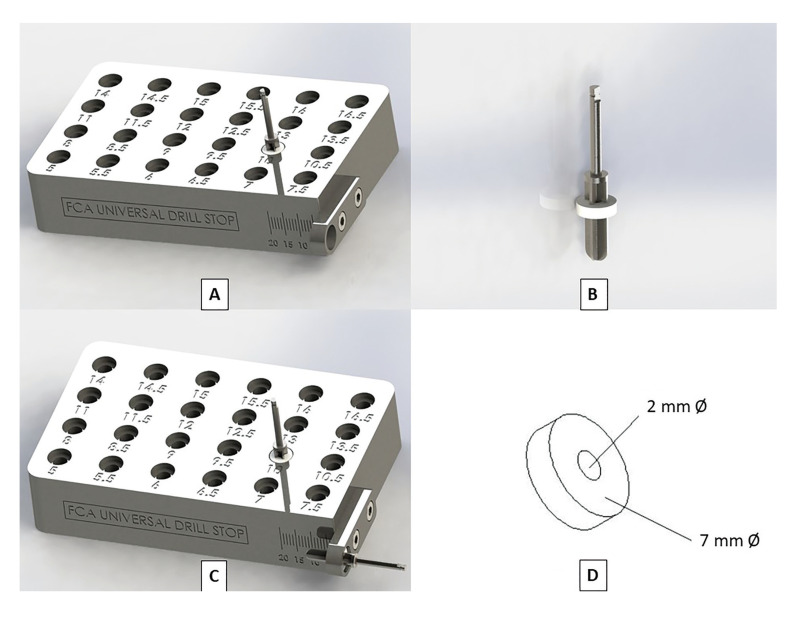
New universal and disposable drill stop device for dental implant drills FCA Universal Drill Stop. A: Stainless steel depth gauge, where a drill stop with a depth limitation of 10 mm is placed. B: Stop placed on the drill, limiting the osteotomy depth to 10 mm. C: Check before starting the osteotomy that the selected depth is 10 mm. D: Disposable PTFE disc-shaped stop with a diameter of 7 mm and a central hole of 2 mm.

Group 3 (drilling with the new universal and disposable stop device for dental implant drills FCA Universal Drill Stop, *n*=100) attached to the drills without stops (Nueva Galimplant® S.L.U., Lugo, Spain) (Fig. 1). This new device was registered as a Utility Model at the Spanish Patent and Trademark Office of the Ministry of Industry, Energy and Tourism of Spain (U202132218) on 10-11-2021. Based on the simple stop devices for endodontic files, this system of stops for implantological drills consists of a sterilisable and reusable depth gauge and disposable drill stops. The depth gauge is a prism made of stainless steel in a numerical control machining centre, with dimensions of 10 x 6.1 x 2 cm. This device has 24 holes on its upper side with a diameter of 4.5 mm at the bottom and 7.3 mm at the top (these dimensions allow clamping and slight expansion of the disposable stops). The 24 perforations allow limiting the depth of the osteotomies from 5 mm to 16.5 mm, and the depth limitation can be increased every 0.5 mm (Fig. 2). In addition, this depth gauge has a device on its front side to check that the selected depth is correct before starting drilling (Fig. 2). Finally, the disposable stops are discs made of polytetrafluoroethylene (PTFE) (Sigma-Aldrich Chemistry, S.A., Madrid, Spain) on a numerical control lathe. PTFE is a polymer like polyethylene, where hydrogen atoms are replaced by fluorine atoms (CF2=CF2). These disposable stops are disc-shaped, 7 mm in diameter and 2 mm thick, with a 2 mm diameter central hole through which 2, 2.6, 3.2 and 3.6 mm diameter drills are inserted (Fig. 2).

- Clinical quantification of the depth of osteotomies

**Figure 3:**
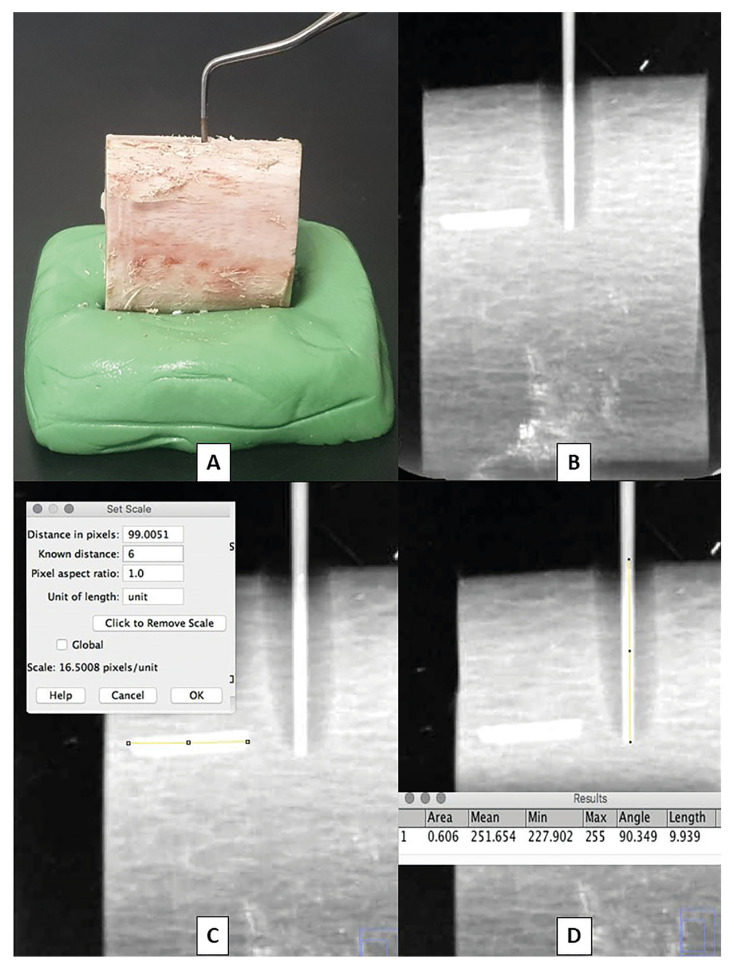
Clinical and radiological quantification of osteotomy depth. A: Clinical measurement of the osteotomy depth using a periodontal probe. B: Digital radiograph of the bone block after inserting a gutta-percha cone into the osteotomy and placing a 6 mm long metal segment on the anterior side of the block to calibrate the radiological images. C: Measurement with ImageJ® of the know distance of the metal segment. D: Measurement with ImageJ® of the depth of the osteotomy.

Both clinical and radiological quantification of the depth of osteotomies in the three study groups was performed by two previously trained and blinded observers, and the arithmetic mean from both was obtained. The clinical depth of the osteotomies was measured in mm using a periodontal UNC probe no. 15 (Hu-Friedy, Chicago, IL, USA) (Fig. 3).

- Radiological quantification of the depth of osteotomies

To facilitate radiological study, bone blocks ≥3 cm in length were removed from their silicone base. Subsequently, a cone of gutta-percha no. 40 (Dentsply Maillefer, Balaigues, Switzerland) was inserted along the entire depth of the osteotomy to facilitate radiographic visualisation, and a 6 mm long metal segment was placed on the anterior side of each block to allow subsequent calibration of the digital radiographic images (Fig. 3). The blocks were deposited on a flat radiographic film and radiographed using a CS 2100 System dental radiography system (Carestrem Health, Inc., Rochester, NY, USA) at 70 Kv, 8 mA, an exposure time of 0.16 sec and a focusing distance of 10 cm. The X-ray beam was perpendicular to the axial axis of the osteotomy and perpendicular to the CS 7600 digital imaging plate (Carestrem Health, Inc., Rochester, NY, USA). Finally, osteotomy depth was quantified using the image analysis software ImageJ® version 1.48v (National Institutes of Health, Maryland, USA) (Fig. 3).

- Statistical analysis

Data were analysed using SPSS 20.0 statistical programme (SPSS® Inc, Chicago, IL, USA). A descriptive study was carried out for each variable. The Kolmogorov-Smirnov normality test and Levene’s test for homogeneity variance were applied, and the data showed a normal distribution and were analysed using parametric tests. The associations between the different qualitative variables were studied using Pearson’s chi-squared test. ANOVA and Tukey tests were applied to the quantitative variables, in each case determining whether the variances were homogeneous. Pearson’s correlation coefficient was used to evaluate the correlation among clinical and radiological measurement of osteotomy depth. Statistical significance was accepted for p≤0.05.

**Figure 4:**
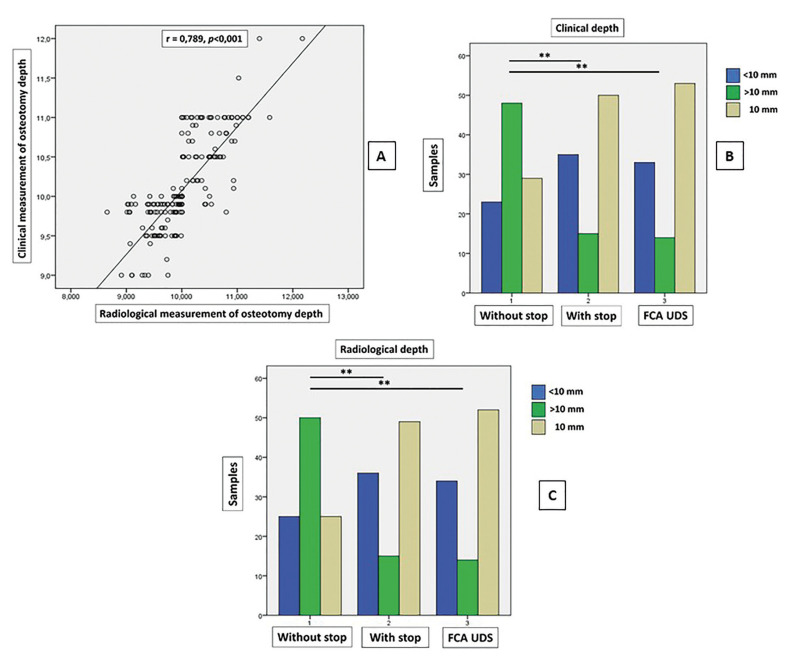
A: Correlation between clinical and radiological measurement of osteotomy depth. B: Comparison between frequency of underdrilled (<10 mm), overdrilled (>10 mm) and exact (=10 mm) measured clinically, significant difference (* p≤0.05; ** *p*<0.001). C: Comparison between frequency of underdrilled (<10 mm), overdrilled (>10 mm) and exact (=10 mm) measured radiologically, significant difference (* p≤0.05; ** *p*<0.001).

## Results

When calculating the correlation between clinical and radiological measurement of osteotomy depth, Pearson's correlation coefficient was r=0.789 indicating a high correlation between the two variables (*p*<0.001) (Fig. 4).

The mean depth of osteotomies performed with prefabricated drills with stop and those performed with FCA Universal Drill Stop were closer to the exact expected depth of 10 mm than those performed without stop, both clinically (drill with stop 10.05 ± 0.38 mm; FCA UDS 9.95 ± 0.34; and drill without stop 10.27 ± 5.91) and radiologically (drill with stop 9.92 ± 0.39; FCA UDS 9.89 ± 0.32; and drill without stop 10.24 ± 0.54); observing statistically significant differences (p≤0.05) between both groups in which stops were used with respect to osteotomies performed without stops (Table 1).

When we classified the 300 osteotomies into underdrilled (<10 mm), overdrilled (>10 mm) and exactly drilled (=10 mm), clinically we observed that the order from highest to lowest percentage of exact osteotomies (10 mm deep) was group 3 (FCA UDS, 53%) > group 2 (drill with stop, 50%) > group 1 (drill without stop, 29%), with statistically significant differences (*p*<0.001) between both groups where stops were used compared to osteotomies performed without stops (Fig. 4).

Radiologically, we also observed that the order from highest to lowest percentage of exact osteotomies (10 mm depth) was group 3 (FCA UDS, 52%) > group 2 (drill with stop, 49%) > group 1 (drill without stop, 25%), with statistically significant differences (*p*<0.001) between both groups where stops were used compared to osteotomies performed without stops (Fig. 4).

## Discussion

The increase in life expectancy, the reduction in the economic costs of implant treatment due to the growth of what is known as “corporative dentistry” (chains of clinics run by single entities that also offer dental insurance plans), and increasing social awareness have together contributed to the growing numbers of implants placed around the world (21), so that in many countries millions of dental implants are placed every year (22). This increase in the number of dental implant surgeries increases the risk of causing iatrogenic injuries to the maxilla when implant osteotomies are performed.

Injuries to the maxillary sinuses and nostrils can occur in the upper maxilla. Accidental perforation of the maxillary sinus mucosa can lead to acute or chronic inflammation, and thickening of the sinus mucosa, as well as the presence of mucosal retention cysts or pseudocysts, can increase the risk of obstruction of the ostium, eventually leading to sinusitis (23). In this regard, in the meta-analysis published by Kim *et al*., in 2019 (7) it was observed that in relation to postoperative sinusitis, among 25 studies and 2301 patients, postoperative sinusitis occurred in 118 patients so the proportion of sinusitis using the single ratio test was 0.05 (95% CI, 0.04-0.07); while regarding intraoperative perforation of the sinus membrane, among 24 studies and 3162 sinuses, perforation of the sinus membrane occurred in 644 sinuses so the proportion of sinusitis using the single proportion test was 0.17 (95% CI, 0.13-0.22). The anterior maxilla is an area of high bone resorption after tooth loss, due to the inclination of the alveolar ridge, so that a much too deep osteotomy in this area can lead to penetration of the implant drills into the nostril, with a high risk of perforation of the nasal mucosa and may even interfere with airflow (9).

In the jaw, iatrogenic trigeminal nerve injuries can result in a neurological deficit ranging from complete loss of sensation (anaesthesia) to a mild decrease in feeling (mild hypoesthesia), altered sensation (dysesthesia), whereby up to 70% of the affected patients may feel pain (24,25). Although the incidence of inferior alveolar nerve (IAN) injury is unknown, probably due to the discrepancy in diagnostic methods used for its diagnosis (diagnosis according to early signs and symptoms reported by patients, based on subjective sensory testing, mechanoceptive methods, nociceptive methods, based on objective sensory testing, and alternative methods) (24), some authors have reported an incidence rate of 40% or more (13-15,26). Regardless of the actual incidence of IAN injury related to dental implant surgery, the signs and symptoms in these patients greatly affect their quality of life: constant, spontaneous, or evoked neuropathic pain; intense postoperative pain not relieved by usual analgesics; functional problems such as interference with speaking, eating, kissing, shaving, applying makeup, tooth brushing, and drinking; psychological problems and depression, numbness/altered sensation due to anaesthesia, hypoesthesia, dysesthesia, or allodynia; or chronic neuropathic pain (15).

To avoid all these potential iatrogenic injuries that can occur during dental implant surgery, some intraoperative risk management strategies have been proposed, such as surgical guides (CBCT-guided static stents have shown to improve the surgical precision of implant placement), safety zone (2-4 mm), use of local anaesthetic (infiltration only), flap design (for better intraoperative vision), use of short implants, replacement of implant drills (manufacturers recommend that implant drills should be replaced frequently, ideally after 10 to 15 cases), intraoperative check x-rays, and use of drill stops (9,27).

However, even though it is known that the use of drill stops increases intraoperative safety by reducing the risk of causing iatrogenic injury, it is estimated that only 11% of clinicians use these devices (13-15). It is difficult to know why these devices are not used more frequently in dental implant surgeries, but it could be because: the lack of universality of these devices as it could make their use difficult for clinicians using different dental implant systems; the small size of these devices which are usually attached onto drills and after surgery taken off the drills for washing and sterilisation, this makes their cleaning and constant use difficult; finally it could be due to the high economic cost of these devices, especially the prefabricated drill systems with stop. For these reasons we have designed the new universal device for disposable drills.

In our study we observed that, both clinically and radiologically, the order of highest to lowest percentage of accurate osteotomies (10 depth) was group 3 (FCA UDS) > group 2 (drill with stop) > group 1 (drill without stop). Our results show the clinical significance of using some implant drill stop system, because drills below 10 mm can mean that the implant is not below the bone and drills above 10 mm can mean nerve injures or sinus membrane damage (7,24,25).

These results point to FCA UDS, despite having the advantages of being a universal device and using disposable stoppers, has enormous accuracy in the depth of implant osteotomies. In addition, another great advantage of this new device is its low cost, because the stops are made of PTFE, which is a very inexpensive polymer. However, despite its low cost, PTFE has ideal physicochemical properties for the manufacture of these stoppers due to its biocompatibility, as it is a practically inert material that does not react with other chemical substances, except in very special situations (28). Finally, it has a low coefficient of friction, is impermeable (maintaining its properties in humid environments such as inside the mouth), has a low dielectric constant, is non-stick, and has unusually high thermal stability and mechanical strength (with a high melting point of 300° C). Because of these properties, PTFE has been widely used in medicine, in the fabrication of vascular and cardiac prostheses, auricular, nasal, and in dentistry as a membrane for bone regeneration, for adhesive cementation of dental restorations, although its most frequent use is the sealing of screw access holes in implant abutments (29).

Our drill stops were manufactured in PTFE because of all the properties described above, and above all because of its toughness and flexibility (29). The strength of this polymer prevents it from breaking and any type of deformation of the stops when inserting the drills into them, and the flexibility allows the central hole of the stops to yield and adapt properly when the diameter of the drill is greater than 2 mm, thus avoiding having to have stops with central holes of different diameters to insert the different diameters of drills available on the market.

Although drill stop systems have not changed in recent years, except for the appearance of prefabricated drills with specific stops for each brand of implant, Schwarz *et al*., in 2015 (30) described a new system of implant drill stops with an eccentric sensor that allowed the drill to stop automatically when the sensor detected soft tissue.

One of the main limitations of the study was that we could not compare the results with other studies, as this is the first study on the depth accuracy of implant osteotomies performed with this new disposable universal stop device.

## Conclusions

In conclusion, the new disposable universal stop system for implant drills FCA Universal Drill Stop, offers similar accuracy to prefabricated drills with a stop, both systems being much more accurate than implant drills without a stop. Although this experimental evaluation showed favourable results, further studies, particularly clinical trials, are needed to demonstrate the efficacy of this new system device.

## Figures and Tables

**Table 1:** Comparison of clinical and radiological measurement of osteotomy depth between study groups (Tukey’s test).

Study groups	n	Clinical depth (mean ± SD)	Radiological depth (mean ± SD)
Drill without stop	100	10,27 ±0,91^#,§^	10,24 ± 0,54^#,§^
Drill with stop	100	10,05 ± 0,38^*^	9,92 ± 0,39^*^
FCA UDS	100	9,95 ± 0,34^*^	9,89 ± 0,32^*^

^* ^Significant difference compared to group 1 (drill without stop); ^# ^Significant difference compared to group 2 (drill with stop); ^§ ^Significant difference compared to group 3 (FCA UDS).
